# A Microfluidic Chip for Detecting Cholangiocarcinoma Cells in Human Bile

**DOI:** 10.1038/s41598-017-04056-2

**Published:** 2017-06-26

**Authors:** Lien-Yu Hung, Nai-Jung Chiang, Wei-Chun Tsai, Chien-Yu Fu, Yu-Chun Wang, Yan-Shen Shan, Gwo-Bin Lee

**Affiliations:** 1 Department of Power Mechanical Engineering, National Tsing Hua University, Hsinchu, Taiwan; 2 National Institute of Cancer Research, National Health Research Institutes, Tainan, Taiwan; 3 Division of Hematology and Oncology, Department of Internal Medicine, National Cheng Kung University Hospital, Tainan, Taiwan; 4 Institute of Clinical Medicine, National Cheng Kung University Hospital, Tainan, Taiwan; 5 Institute of Biomedical Engineering, National Tsing Hua University, Hsinchu, Taiwan; 6 Institute of NanoEngineering and Microsystems, National Tsing Hua University, Hsinchu, Taiwan

## Abstract

Cholangiocarcinoma (CCA), a biliary tract malignancy, accounts for 20% of all liver cancers. There are several existing methods for diagnosis of CCA, though they are generally expensive, laborious, and suffer from low detection rates. Herein we first developed a means of partially purifying human bile for consequent injection into a microfluidic chip. Then, the novel microfluidic system, which featured 1) a cell capture module, 2) an immunofluorescence (IF) staining module featuring two CCA-specific biomarkers, and 3) an optical detection module for visualization of antibody probes bound to these CCA marker proteins, was used to detect bile duct cancer cells within partially purified bile samples. As a proof of concept, CCA cells were successfully captured and identified from CCA cell cultures, blood samples inoculated with CCA cells, and clinical bile specimens. In 7.5 ml of bile, this system could detect **>**2, 0, and 1 positive cells in advanced stage patients, healthy patients, and chemotherapy-treated patients, respectively. In conclusion, our microfluidic system could be a promising tool for detection of cancer cells in bile, even at the earliest stages of CCA when cancer cells are at low densities relative to the total population of epithelial cells.

## Introduction

**Figure 1:**
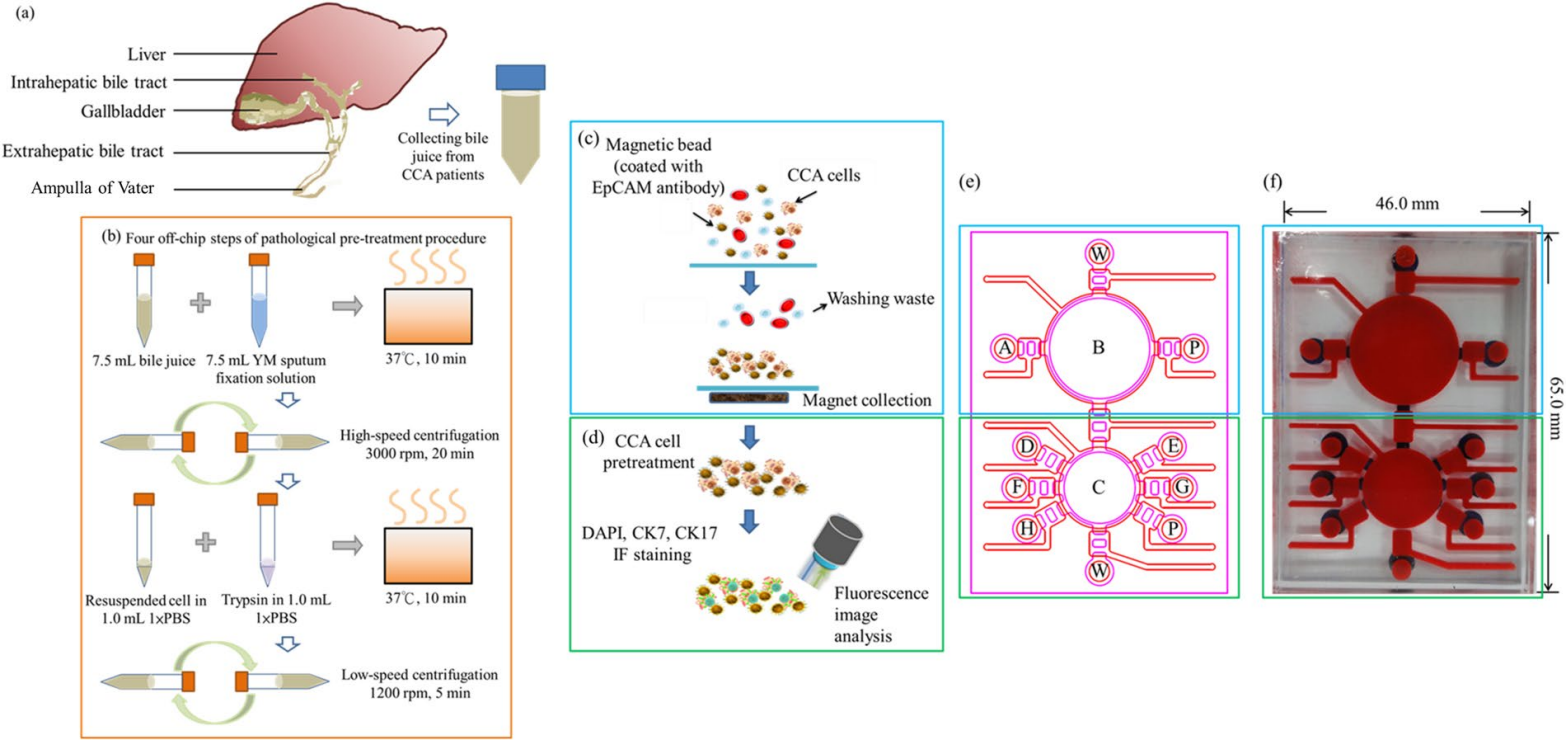
(**a**) Illustration of the relative positions of the liver, gallbladder, and biliary tree. (**b**) The working process of the four off-chip pre-treatment steps for preparation of bile. (**c**) Illustration of on-chip cholangiocarcinoma (CCA) cell capture, washing, and collection. (**d**) Illustration of on-chip immunofluorescence (IF) staining and analysis. Please see the main text for full names of the molecular probes/antibodies. (**e**) The integrated microfluidic chip equipped with the following micro-devices: A = sample loading chamber, B and C = membrane-type micromixers/micropumps, D = 4% paraformaldehyde chamber, E = 0.1% Triton X 100 chamber, F = first antibody chamber, G = secondary antibody chamber, H = DAPI/Hoechst stain chamber, P = PBS (1x) chamber, and W = waste outlet; (**f**) A photograph of the chip. The red ink indicates the air control layer, and the blue ink represents fluid channel layer.

Cholangiocarcinoma (CCA) is a rare and lethal cancer that arises from bile duct epithelial cells (Figure 1a)^1–3^. CCA can be clinically classified as either “intrahepatic” (IH-CCA) or “extrahepatic” (EH-CCA), and each type requires its own unique treatment plan; in the former, cancer cells originate from the bile duct within the liver, and 10–20% of primary liver tumors form this way^4–6^. More than half of all CCA-positive patients have EH-CCA, the most common type of CCA^3, 7^.

Several methods exist for diagnosis of CCA, including blood tests^8^, abdominal sonography^9^, computed tomography scans^3^, magnetic resonance imaging^3^, and endoscopic retrograde choledochopancreatography^9^. However, the biomarkers targeted in blood tests differ little from those assayed during liver function tests. For instance, carcinoembriogenic antigen (CEA) and carbohydrate antigen 19-9 (CA19-9) are commonly used biomarkers^8^, though they are not uniquely expressed bile duct cancer cells. Furthermore, CCA is difficult to diagnose in its early stages with existing bio-imaging methods, since the gallbladder and biliary tracts are deep inside the abdomen. Therefore, most CCAs are diagnosed in their later stages by endoscopic methods such as laparoscopy and cholangioscopy; however, these common, painful processes are characterized by low detection rates and require expensive instrumentation and large biopsies^3, 9^.

Histological analyses are crucial for diagnosis of CCA; such approaches cannot only distinguish between IH-CCA and EH-CCA, but they also aid in estimating how advanced the cancer is^10, 11^. A previous work investigated biliary cytology with 100 patients and separated them into two groups, including group 1 for bile cytology test only and group 2 for combining bile and brush cytology tests to diagnosis whether they were invasive by cancer^12^. Experimental data showed that cancer cells could be found in bile juice and could be used for diagnosis. Sensitivities in group 1 and group 2 were 33% and 69%, respectively. However, it is often difficult to assess and treat patients in the early stages of CCA given the low sensitivity of such histology-based approaches. For example, standard cytological tests performed with bile tract brushings only diagnosed CCA in 50% of CCA-positive samples^10, 13^. Combining cytological with histological approaches, however, may increase the detection rate to 70%^7, 14^. Furthermore, fluorescence *in situ* hybridization assay was also combined with biliary brushing specimens for biliary cancer analysis, with 91% of specificity^15^. However, the sensitivity was only 34% and it took a relatively lengthy process. Nevertheless, at the present time, most patients are diagnosed so late that prognoses are typically poor.

Recently, detection of circulating tumor cells (CTCs) has been recognized as a valid approach for cancer diagnostics, particularly bile duct cancers^16^. When using the relatively expensive CELLSEARCH^®^ system, 25% of tested patients with CCA were characterized by possession of two or more detectable CTCs in 7.5 mL of human blood^17^. The biomarkers used by CELLSEARCH^®^ are the epithelial cell adhesion molecule (EpCAM), cytokeratins (CK) 8, 18, and 19, though they are not specific to CCA. In contrast, CK7 and CK17 are over-expressed by CCA cells, and antibodies can be used to label these proteins *in situ* ^18^. Similarly, tumor cells over-expressing EpCAM have been applied to capture them from liquid biopsy by using multiple techniques, and EpCAM has also been found over-expressed in cholangiocarcinoma and gallbladder cancer^17^. Thus we hypothesized that CCA cells in bile could be captured and might correlate with the stage of disease.

In general, it is difficult to detect cancer cells in bile, and false positive and negative results can occur^14, 15^. It is therefore of critical importance to develop new technologies for detecting CCA within human bile. Recently, microfluidic techniques have emerged as promising tools for detection of cancer cells^16, 19–21^, and they possess several advantages over their large-scale counterparts, including compactness, portability, high sensitivity, low consumption of samples and reagents, automation, and integration. Therefore, we combined a bench-top bile processing method (Figure 1b) with a microfluidic system (Figure 1c) to screen human bile for the presence of the CCA biomarkers EpCAM, CK7 and CK17. This rapid (35 min) system featured a cell capture module, an immunofluorescence (IF) staining module, and an optical detection module and successfully identified CCA cells in low abundance; it could therefore be a promising tool for CCA diagnostics.

## Results

### Experimental procedure and chip design of on-chip CCA diagnosis and prognosis

As shown in Figure 1, the whole working process was composed of four off-chip steps of pathological pre-treatment procedure to bile specimens, one on-chip cell capture process and one on-chip IF staining process. The relative positions of liver, gallbladder and biliary tree are shown in Figure 1(a). The four off-chip steps of pathological pre-treatment procedure were shown in Figure 1(b). Briefly, 7.5 mL of bile was mixed with 7.5 mL YM sputum fixation solution and incubated at 37 °C for 10 minutes. Then a high-speed centrifugation step to remove mucin-liked substrates and gallbladder sludges was performed. Cell pellets were then resuspended in 1x phosphate-buffered saline buffer (PBS) and treated with 1x trypsin. Then trypsin treated solution was diluted, and a low-speed centrifugation step was used to collect cells. Cell pellets were resuspended in 1x PBS buffer and loaded into the sample buffer loading chamber A of the microfluidic chip (Figure 1(c)). The on-chip process started from the incubation between epithelial-enrich immunomagnetic beads (EpiEnrich beads, anti-EpCAM antibodies coated beads) and the cell pellets to capture the target cancer cells in bile (Figure 1(b)). After magnetic collection and washing out unbounded materials (Figure 1(c)), the bead-cell complexes were transported from the cell capture module to the IF staining module (Figure 1(d)). Before IF staining, captured cells were fixed by paraformaldehyde and permeabilized by triton X-100, for the properly staining of intra-cellular cytokeratin (CK)-related biomarkers. The first antibodies (Abs) of CK7 and CK17 were then applied to incubate with bead-cell complexes for 30 minutes. After magnetic collection and washing out unbounded first Abs, fluorescence-labelled secondary Abs for CK7 and CK17 were applied to incubate for another 5 minutes. DAPI (4′, 6-diamidino-2-phenylindole) or Hoechst, the nucleic staining dyes, were then transported from a storage chamber and incubated with the cells for one minute. After the final magnetic collection and washing out secondary Abs and nucleic staining dyes, the bead-cell complexes were ready to be detected by the optical detecting module. The detailed operating process of the on-chip CCA diagnosis and prognosis could be found in Supplemental Information Table 1. It is worth noting that all the stages of on-chip incubation, transportation, and washing, with the exception of sample and reagent loading, were performed automatically in the microfluidic chip by activating micropumps, micromixers and microvalves. When compared with the Abs incubation steps of bench-top IF staining process, the experimental time of on-chip tests could be greatly reduced from 90 minutes to 35 minutes^22, 23^. Detail information about the micromixer/micropump, and microvalves could be found in Supplemental Information.

### Capture and immunofluorescence (IF) staining of CCA cell line by using the microfluidic system

**Figure 2:**
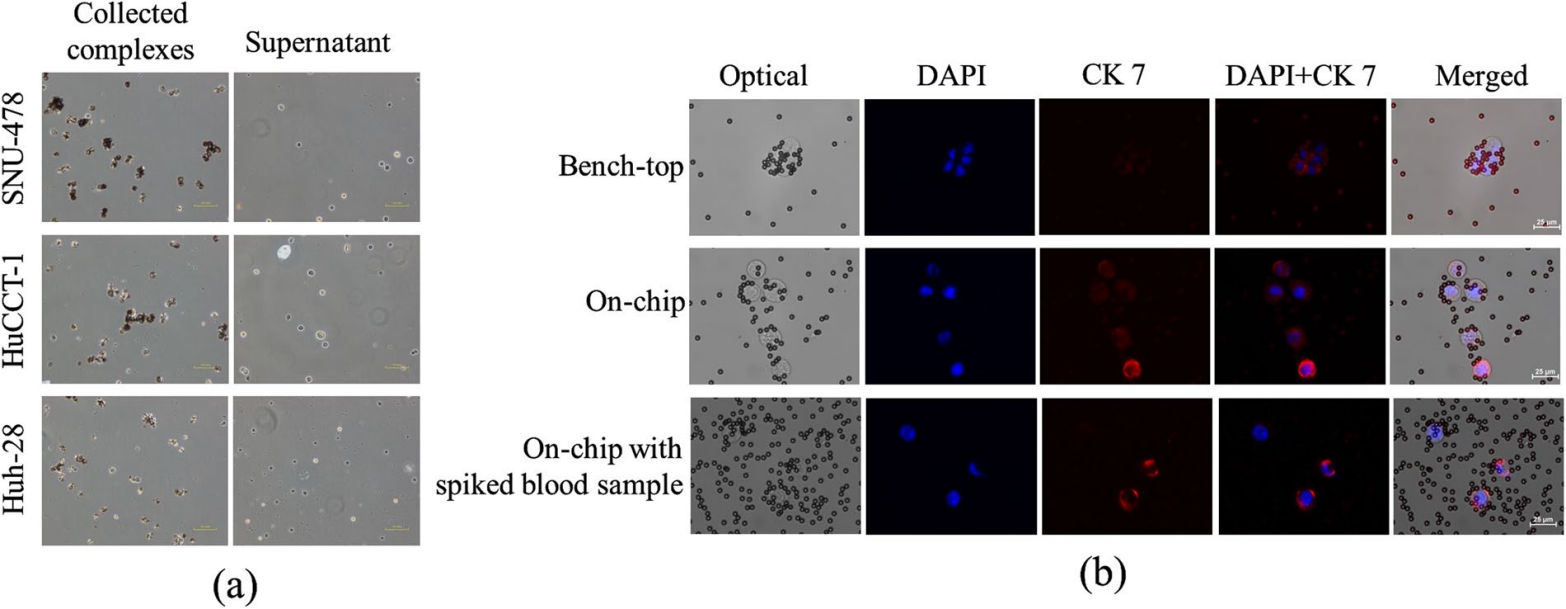
(**a**) On-chip cell capture was tested with three cholangiocarcinoma (CCA) cell lines- SNU-478, HuCCT-1, and Huh-28. (**b**) On-chip immunofluorescence (IF) staining was tested with SNU-478 cells (bench-top and on-chip), and SNU-678-spiked blood samples (on-chip only).

Three CCA cancer cell lines were tested on-chip to determine whether such epithelial cells could be recognized by EpiEnrich beads; such was indeed the case, and on-chip capture of SNU-478, HuCCT-1, and Huh-28 cells were 91.2 ± 2.2 (SD for this and all error terms henceforth), 84.4 ± 1.5, and 80.6 ± 3.1%, respectively. Since SNU-478 cells were most readily captured (Figure 2a), we compared the efficacy of cell capture for (1) the bench-top and (2) on-chip protocols with SNU-478 cells in PBS, and (3) the on-chip protocol with SNU-478-inoculated human blood. The bench-top protocol could not stain SNU-478 cells effectively, and only a weak fluorescent signal was observed (Figure 2b). In contrast, SNU-478 cells were stained clearly on-chip with the CCA biomarker CK7 (Figure 2b). SNU-478-inoculated blood samples yielded a similar CCA cell capture rate (around 85%) as for SNU-478 cells only (data not shown), and the fluorescence signal intensities of both CK7 and DAPI appear similar to those of pure CCA cells.

### Partial purification of human bile and PAP staining results

**Figure 3:**
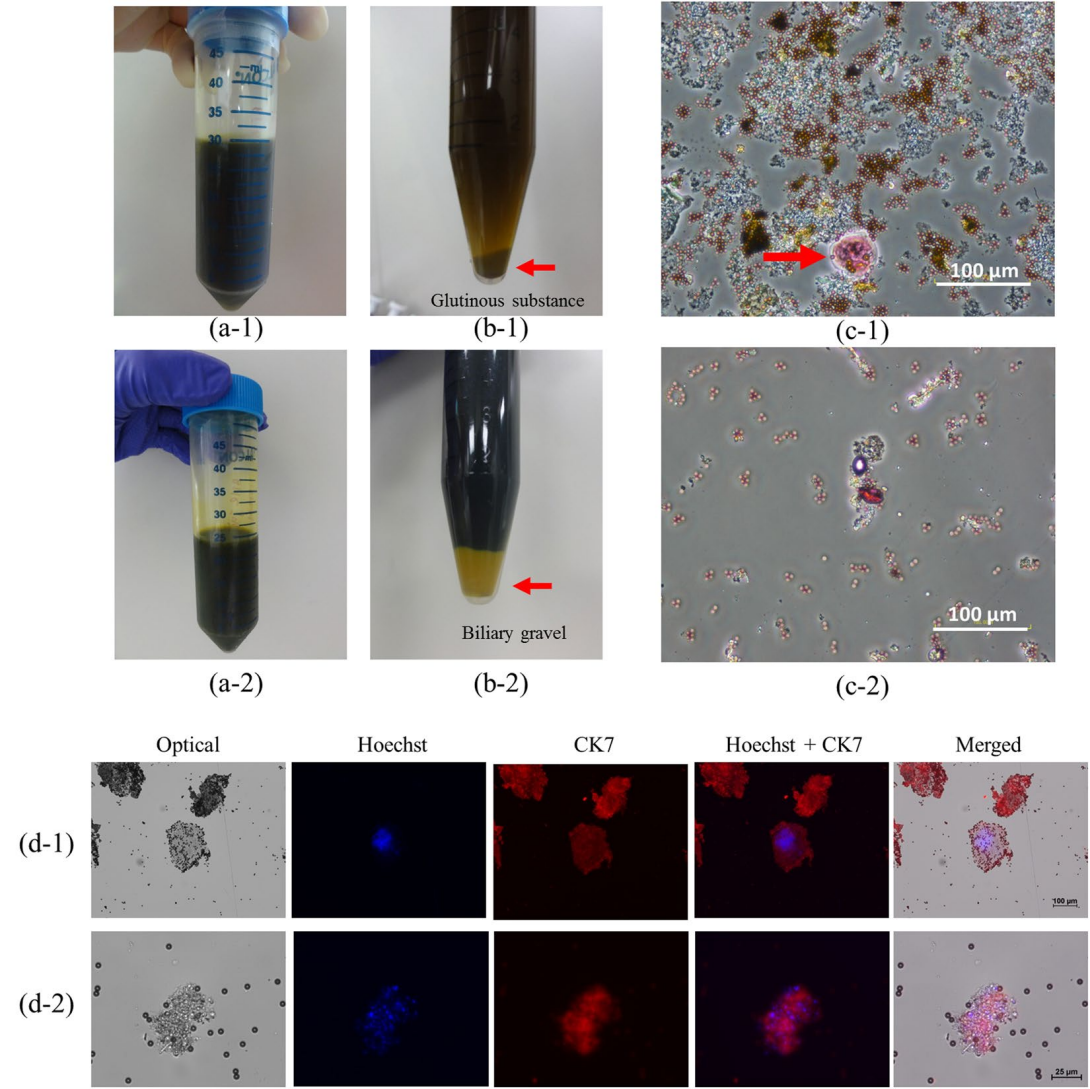
(**a**) Bile specimens before and (**b**) after partial bile epithelial cell purification. (**c**–**d**) Bile specimens from cholangiocarcinoma (CCA) patients tested using a bead capture-based bench-top protocol followed by PAP staining. (a-1 to d-1) a bile specimen of liver hilar tumor/CCA (stage IV). (a-2 to d-2) a bile specimen of intrahepatic CCA (stage III).

Clinical bile samples varied in color and consistency (Figure 3a1–2). After centrifugation, glutinous substances and biliary gravel were pelleted (Figure 3b1–2). The resuspended pellets were then concentrated again via centrifugation using a cytospin procedure to relocate bead-cell complexes onto glass slides for Papanicolaou (PAP) staining, though glutinous substances and biliary gravel were still observed (Figure 3c1–2). Despite the visible presence of such contaminants, the fluorescent staining signals for the CCA biomarker CK7 were still quite strong for two CCA patient bile samples (Figure 3d1–2).

**Figure 4:**
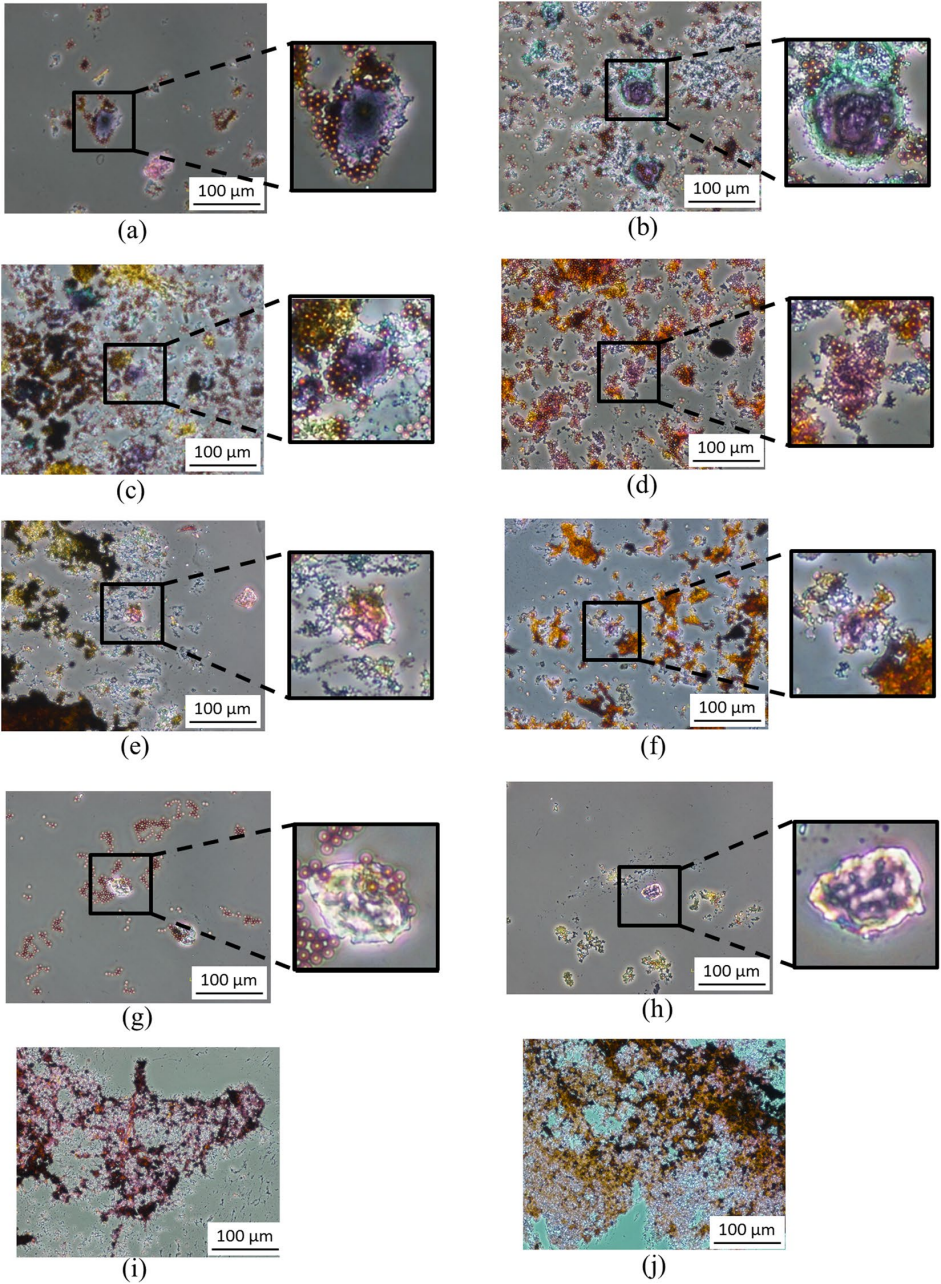
Bile specimens from eight cholangiocarcinoma (CCA) and two negative control patients analyzed by a bench-top bead-capture protocol followed by PAP staining. (**a**) Liver hilar tumor/CCA (stage IV). (**b**) CCA with lung metastases (stage IV). (**c**) Gallbladder cancer with lung metastases (stage IV). (**d**) Recurrent CCA (liver metastasis; stage IV). (**e**) Intrahepatic CCA (stage III). (**f**) CCA with peritoneal carcinomatosis (stage IV). (**g**) Malignant neoplasm of intrahepatic bile ducts (stage III). (**h**) CCA (stage IIIb, after chemotherapy). (**i–j**) Non-cancer patients (cholecystitis).

Figure 4(a–h) shows the PAP staining results for CCA patients 1–8. The CCA cancer cells could be observed from the PAP staining cyto-sections, and no such cells were observed in the negative controls (Figure 4i–j). All sections contained plentiful debris (some of which were stained). Although it is likely that only a trained pathologist could interpret these findings and make an accurate diagnosis, a reduction in background noise could generate a more readily interpretable staining pattern.

### On-chip cancer cell capture and IF staining

**Figure 5:**
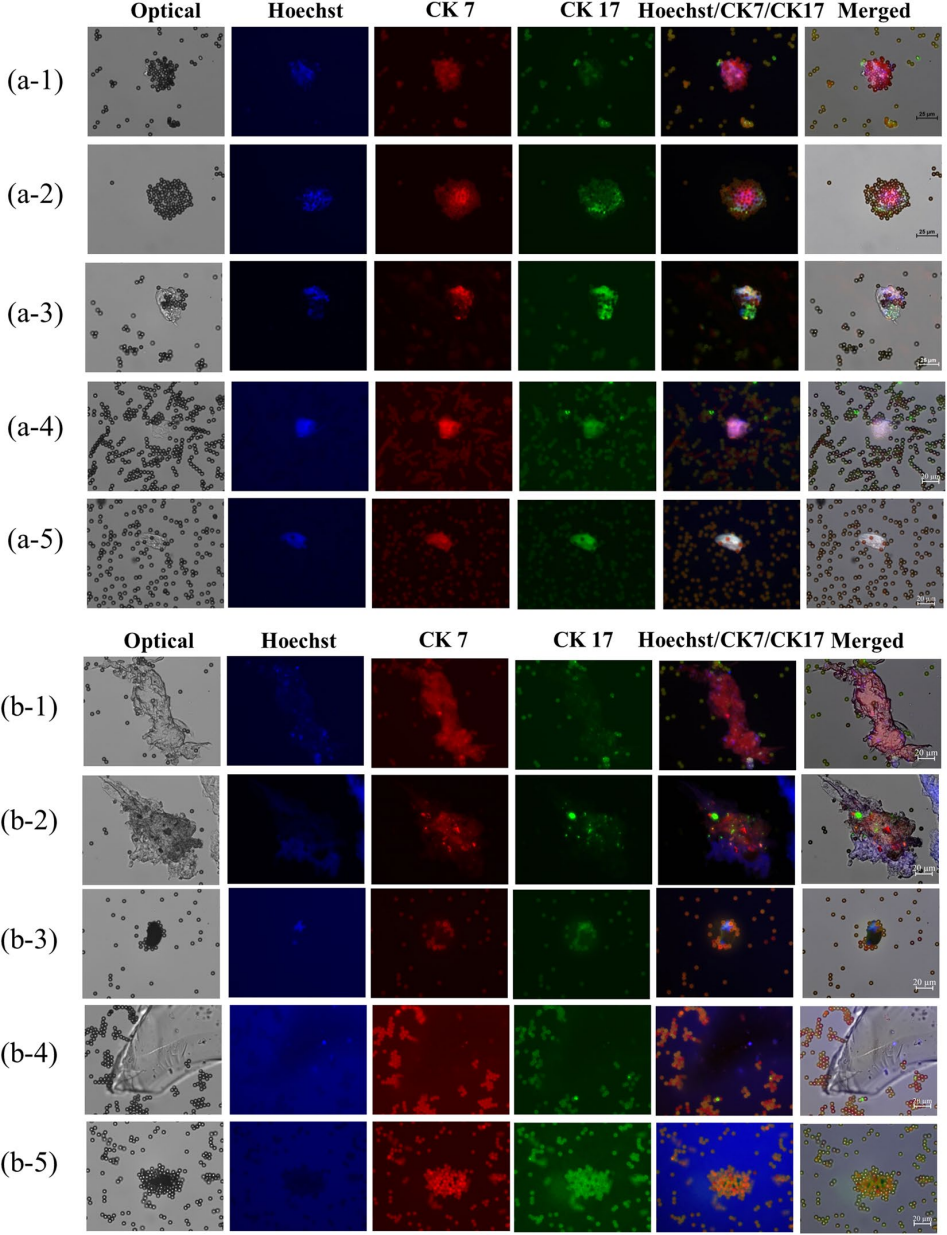
On-chip cholangiocarcinoma (CCA) cell capture and consequent immunofluorescence (IF) staining with CCA-specific biomarkers (CK7 and CK17). Cells were additionally stained with EpCAM and DAPI/Hoechst. Please note that only cells that presented red (CK7) green,(CK17), and blue (DNA) fluorescence were confirmed to be CCA cancer cells that were captured by EpiEnrich beads. (**a**) Positive CCA cells; (**b**) negative samples.

On-chip cell capture and IF staining steps were performed on the developed microfluidic system in an automated manner. The micromixers, micropumps, and microvalves collectively worked to ensure that CCA cells were captured by magnetic beads and consequently labelled with antibodies. The micropumps/micromixers generated a well-controlled shearing force that effectively washed out glutinous substances and biliary gravels; such contaminants were commonly observed in the bench-top procedures (Figure 3). The on-chip CCA cancer cell capture protocol which required pre-processing of bile samples were performed with large (2 × 10^5^) and small amounts (10 cells and one cell) of spiked-in cells, which presented 82.5 ± 4.3, 70.0 ± 8.1, and 66.6 ± 4.7%, respectively, as shown in Supplemental Information Figures 2 and 3, Supplemental Information Tables 2, and 3. It is important to note that these cell-spiked bile fluids were purified from diseased-bile samples by centrifugation since there was no non-diseased bile sample available. This reduction in background noise allowed for better IF staining of CCA cells with target biomarker probes (CK7 and CK17) under a fluorescent microscope (Figure 5a). Although some particles were stained by CCA biomarkers in the “negative” (i.e., non-CCA) cells (Figure 5b), these samples were too small to represent actual cells (<20 μm); accordingly, they did not present nucleic acid staining patterns. In other negative controls (e.g., Figure 5b3), some cell-sized particles (~20 μm) were not stained by any of the three markers, as hypothesized.

Bile samples from eight CCA patients were tested using the developed microfluidic system, and the number of positive CCA cancer cells and negative particles were quantified and averaged (Table 1). Patients at the later stages of CCA had two or more positive CCA cells on average, in contrast to no such cells being quantified in non-cancer patients. It is worth noting that one patient being treated with chemotherapy also possessed a small number of positive cells (two positive cells across three repeated experiments).

**Table 1:** Number of cholangiocarcinoma (CCA; “positive”) and non-CCA (“negative”) cells captured from two technical replicates (i.e., bile aliquots) from each of eight CCA patients with the on-chip CCA diagnostics system.

Patient No.	Diagnosis	Captured positive cells (#)	Captured negative cells (#)
01	Liver hilar tumor/CCA (stage IV)	3.6 ± 1.3	5.4 ± 3.7
02	CCA with lung metastases (stage IV)	1.3 ± 1.5	2.3 ± 1.5
03	Gallbladder cancer with lung metastases (stage IV)	3.0 ± 1.4	2.5 ± 0.7
04	Recurrent CCA (liver metastasis; stage IV)	2.8 ± 1.3	3.0 ± 2.5
05	Intrahepatic CCA (stage III)	3.3 ± 1.5	2.3 ± 2.1
06	CCA with peritoneal carcinomatosis (stage IV)	3.5 ± 0.7	0.0 ± 0.0
07	Malignant neoplasm of intrahepatic bile ducts (stage III)	2.3 ± 2.3	1.0 ± 0.0
08	CCA stage IIIb (after chemotherapy)	0.0 ± 0.7	1.0 ± 0.8
09	Non-cancer patient (cholecystitis)	0.0 ± 0.0	2.0 ± 0.5
10	Non-cancer patient (cholecystitis)	0.0 ± 0.3	1.3 ± 0.1

Error bars represent standard deviation.

## Discussion

Herein we have shown that the developed microfluidic system can successfully detect CCA cells in bile. This represents the first time that bile has been used directly for rapid CCA diagnosis; such was achieved by partially removing contaminants from the bile in a four-step, off-chip process prior to injection of the sample material into the microfluidic chip^13, 19, 24^. Otherwise, micro-channels might become clogged by the glutinous substances and biliary gravels. After such partial purification of the bile, CCA epithelial cells were captured and incubated with antibody biomarkers for CCA, namely CK7 and CK17. The developed protocol could detect two positive CCA cancer cells in a volume of 7.5 ml, representing the most sensitive CCA diagnostic system ever employed for use with human bile.

The number of captured positive CCA cells was found to be directly proportional to the stage of the CCA cancer, with more CCA cells detected in patients at later cancer stages. For instance, three positive CCA cells were typically detected in those at the later stages of CCA, and less than one was measured in a patient undergoing chemotherapy; the latter results suggests that this system could not only be used for initial diagnosis of CCA, but also to monitor the efficacy of chemotherapy.

It has proven challenging to find a highly specific biomarker that is expressed by only one type of cancer cell, and not by other cell types. Therefore, multiple genes/proteins are typically targeted (two in the case herein) in cancer diagnostics^19^. For instance, CELLSEARCH^®^ exploits several “positive” biomarkers, including EpCAM, CK8, CK18, and CK19, as well as a negative marker (CD45) that reflects contamination with white blood cells^17^. In this study, CK7 and CK17 were found to exhibit high specificity and sensitivity towards CCA cells, though the developed microfluidic system could be readily modified to include additional, or alternative, molecular markers that are later found to be expressed strictly by CCA cells. Similarly, the chip could be modified to target entirely different cancer cells.

The fluorescent images of Figure 3(d-1) were further characterized quantitatively. It contained three pieces of particles presenting red fluorescence signals with only one particle presenting Hoechst signal. This image indicated that there were more than 60% (two out of three particles) non-specific signals presented under bench-top IF staining process. Comparing with the fluorescent images of Figure 5(a-1) to (a-5), all captured cells presented fluorescence signals of three specific biomarkers and no non-specific signals were observed. These results indicated that this microfluidic system could reduce more than 60% of non-specific signals. Furthermore, we provided more fluorescent images presented under the bench-top IF staining process, as shown in Supplemental Information Figure 3. These fluorescent images contained plenty of glutinous substances and biliary gravels, spreading more than 80% area.

Not only is the developed system flexible in terms of molecular targets, but, as mentioned above, it also typically involve less human intervention and is characterized by shorter operating times than their large-scale counterparts. For instance, IF staining time was only 35 min on-chip herein, as opposed to 90 min with the bench-top protocol. The microfluidic system was also able to more effectively wash away non-specific particles, such as biliary gravels, via micropumps and microvalves. Such advantages over bench-top protocols are likely to translate into more accurate CCA diagnoses. Finally, when compared with the commercially available CELLSEARCH^®^ system, the microfluidic CCA diagnostics system is more flexible and could be used to not only detect bile duct cancer cells at low densities, but it could also be adapted to capture and identify other cancer cells.

## Materials and Methods

### Chip design and fabrication

In order to rapidly perform cancer cell capture and IF staining in an automated manner, an integrated microfluidic chip was designed and fabricated. It consisted of two polydimethylsiloxane (PDMS, Sylgad 184 A/B, Dow Corning, USA) layers and a glass substrate (G-Tech Optoelectronics, Taiwan). A computer-numerical-control (CNC) machining process and a PDMS replication process were used to generate a polymethyl methacrylate (PMMA) template that functioned as the master mold for the PDMS structures^25^, and oxygen plasma treatment was used to bond the PDMS layers to the glass substrate (Figure 1f).

Two transportation units (micropump/micromixer)^25^, eight reagent loading chambers, microvalves (which are normally closed)^26^, and two waste outlets were integrated into the chip (Figure 1e). Specifically, two suction-type transportation units were adapted to transport and incubate samples and reagents. Detailed information about these micro-components can be found in a previous work^27^. In brief, these micro-devices were connected to an electromagnetic valve (EMV) that was driven by a custom-made, digital controller connected to a vacuum pump; the vacuum force generated then deflected a thin PDMS membrane, which in turn drove the transport of samples and reagents. Note that in addition to micropumping, the suction-type transportation unit could be used as a micromixing device for incubation of the magnetic beads and CCA cells (Figure 1c) if operated in a different mode^27^. All methods were performed according to the relevant guidelines.

### Biological material

Three types of CCA cells were tested to verify the performance of the developed microfluidic system for on-chip CCA diagnosis. SNU-478 cancer cells isolated from the ampulla of Vater (the interface of the pancreatic and bile ducts) represented the first type^28^. HuCCT-1 cell lines derived from metastatic cancer cells from the ascites of CCA patients were also used as test material^29^. Finally, cells of the Huh-28 cancer cell line were tested; these cells were directly separated from the bile duct close to the liver^30^. All cells were obtained from the Institute of Clinical Medicine of the National Cheng Kung University Hospital (NCKUH) and cultured in Roswell Park Memorial Institute (RPMI) 1640 media (RPMI 1640, Gibco, Invitrogen, USA) containing 2 mM L-glutamine (L-Gln, Gibco), 10% fetal bovine serum (FBS, Gibco), 100 U/mL penicillin, and 100 μg/mL streptomycin (Pen-Strep, Gibco), at 37 °C under 5% CO_2_. These cells were analyzed both on-chip and using a traditional bench-top protocol.

Since the developed system was found to best detect SNU-478 cells, an additional test was conducted to attempt to capture SNU-478 cells spiked into whole human blood at low densities. Blood was inoculated with 2 × 10^5^ SNU-478 cells (n = 3 replicates for spiked cell count). Then, 600 μL of SNU-478 cell-inoculated blood were injected into the microfluidic chip and analyzed as described below.

Clinical bile samples from eight CCA patients (n = 3 technical replicates/sample) were collected and analyzed both on-chip and using the traditional bench-top approach to further validate the efficacy of the developed system. A description of the biopsies can be found in Table 1, and bile was also collected from two non-cancer patients (cholecystitis) to serve as negative controls. All whole blood samples and clinical bile samples were collected and provided by the Institute of Clinical Medicine of NCKUH, and their dissemination was approved by the institutional review board (IRB) of NCKUH under IRB approval # A-ER-103-063. Note that this study used medical residual samples for tests, which was permitted by the IRB of NCKUH to waive informed consent. All methods were performed following the relevant guidelines and IRB regulations.

### Treatment of human bile for CCA chip analysis

Although CCA cells could be directly injected into the chip for binding onto cell-capture beads, four off-chip, pre-treatment steps were needed to prepare unprocessed bile for injection into the chip (Figure 1b). Bile (7.5 mL) was mixed with 7.5 mL YM sputum fixation solution (Muto Pure Chemicals, Japan) and incubated at 37 °C for 10 min. Then, samples were spun at 3000 rpm for 20 min to pellet cells, and the pelleted cells were resuspended in 1 mL of 1x PBS, treated with 1x trypsin in a final volume of 2 mL, and incubated at 37 °C for 10 min. This solution was diluted to 8 mL with additional 1x PBS, and samples were centrifuged at 1200 rpm for 5 min. Cell pellets were resuspended in 600 μL 1x PBS and loaded into sample loading chamber A of the microfluidic system (Figure 1c).

“Epithelial Enrich” immuno-magnetic Dynabeads® (EpiEnrich, anti-EpCAM antibody-coated, 4 × 10^8^ beads/mL, Ø = 4.5 μm, Invitrogen) were used to capture epithelial cells injected into the chip (Figure 1b). In brief, 100 μL of the EpiEnrich beads were washed twice in 1 mL of 1x PBS and re-suspended in 1x PBS in a total volume of 1 mL. Next, 10 μL of EpiEnrich beads were loaded with test cells to generate cell-bead complexes at an approximate ratio of 10^5^ cells: 100 μL beads for the cell line-based verification process. The binding ratios were verified by microscopy^19^.

After magnetic collection and washing out of unbound materials (Figure 1c; see our prior work with this same chip for details^28^.), the bead-cell complexes were transported from the cell capture module to the IF staining module (Figure 1d). Prior to IF staining of intracellular CK biomarkers, cells were fixed in 4% paraformaldehyde for 5 min and permeabilized with 0.1% Triton X-100 (Sigma, USA) for 5 min on-chip. The primary CK7 (N-20; 100 μL, 200 µg/mL, sc-17116, Goat, Santa Cruz Biotech., USA) and CK17 (100 μL, 0.6 mg/mL, GTX103765, Rabbit, GeneTex, USA) antibodies (Abs) were then incubated with bead-cell complexes for 30 min. After magnetic collection and washing out of unbound Abs, fluorescence-labelled secondary Abs for CK7 (donkey anti-goat IgG phycoerythrin (PE); Santa Cruz Biotech.) and CK17 (goat anti-rabbit IgG Alexa 488; GeneTex) were incubated with samples for 5 min. DAPI (4′, 6-diamidino-2-phenylindole) or Hoechst 33342 (Invitrogen) nucleic acid stains were then transported from a storage chamber and incubated with the cells for 1 min. After the final magnetic collection and washing out of secondary Abs and nucleic acid stains, the bead-cell complexes were ready to be detected by the optical detection module, which has been described in a prior work^28^. All aforementioned steps (i.e., Ab incubation, transport, and washing), with the exception of sample and reagent loading, were performed automatically in the microfluidic chip by activating micropumps, micromixers, and microvalves^28^. Only 35 min were required for the on-chip protocol, as opposed to 90 min with the benchtop protocol. All methods were performed according to the relevant guidelines.

### Bench-top protocol for IF-based detection of two CCA biomarkers

As a comparison to the microfluidic chip for CCA diagnosis, pelleted cells from 1) CCA cell lines, 2) blood samples spiked with SNU-478 cells, and 3) clinical bile samples (processed as described above to partially purify the bile prior to injection) were fixed as described above, washed twice with 1x PBS for 5 min, and blocked with 1x PBS containing 3% bovine serum albumin (BSA, Sigma-Aldrich). After removing the blocking buffer, 1000-fold-diluted primary Ab (the same as used on-chip) were incubated with the cells for 60 min at room temperature. After washing out unbound primary Abs, 500-fold-diluted secondary Abs (the same as used on-chip) were added to the samples and incubated for 30 min. Then, the nucleic acid stains DAPI or Hoechst were diluted 5000-fold and used to stain the cells for 5 min. After washing out the unbound secondary Abs and dyes, ProLong® Gold Antifade Reagent (Invitrogen) was used to embed the cells on slides.

The slides were optically analyzed under one collimation lens, an objective lens (Nikon LU Plan 10×/0.30 A, Nikon, Japan), three fluorescent filters (Nikon U-FUW, U-MWIBA2, and UMWIG2), and a mercury lamp (MODEL C-SHG1, Nikon). Images of antibody fluorescence were then captured using a DS-Qi1Mc camera (1.5 megapixels, equipped a Peltier cooling device and a programmable gain amplifier, Nikon) coupled to an inverted microscope equipped with a digital control module^31^. All optical and fluorescence images were captured using NIS-Elements Basic Research software (Br, version 4.20.00, 64 bit, Nikon, Japan).

As another comparison to the microfluidic system, 500 μL of bile from each of the eight patients (Table 1) were treated as described above on a slide, and a modified PAP staining protocol was then undertaken. Briefly, cells were sorted using the Shandon Cytospin^®^ 4 cytocentrifuge (Thermo Fisher Scientific, USA) at 500 rpm for 3 min as described in a previous work^32^. Then, PAP staining was performed in a chemical fume hood using the following solutions in series^33^: 95% ethanol for 30 min, water for 5 min, Gill’s hematoxylin (Muto Pure Chemicals) for 5 min, 0.5% HCl for 5 s, water for 5 min, 95% ethanol for 15 s (twice in two different staining tanks), orange greenish 6 (OG-6, Muto Pure Chemicals) for 80 s; 95% ethanol for 15 s (twice in two different tanks), eosin azure-50 (EA-50, Muto Pure Chemicals) for 5 min, 95% ethanol for 15 s (twice in two different tanks), 100% ethanol for 15 s (thrice in three different tanks), and xylene for 1 min (thrice in three different tanks). Finally, cover slips were placed over all slides. After the bile samples were stained, the cytospin-slides were carefully examined for indications of oncogenesis, and the ensuing diagnostic results were compared with those obtained from the integrated microfluidic system. All methods were performed according to the relevant guidelines.

## Electonic Supplementary Material

Supplemental information
