# Supplementary material for: A Microfluidic Chip for Detecting Cholangiocarcinoma Cells in Human Bile
Source: Sci Rep. 2017 Jun 26;7:4248. doi: 10.1038/s41598-017-04056-2 (PMC5484672; doi:10.1038/s41598-017-04056-2)
Supplement: Supplementary file 1 — Supplemental information [file 41598_2017_4056_MOESM1_ESM.doc]

Supplemental information

**A MICROFLUIDIC CHIP FOR DETECTING CHOLANGIOCARCINOMA CELLS IN HUMAN BILE***

Lien-Yu Hung^1†^, Nai-Jung Chiang^2,3‡^, Wei-Chun Tsai^1^, Chien-Yu Fu^1^, Yu-Chun Wang^4^, Yan-Shen Shan^4+^, and Gwo-Bin Lee^1,5,6*^

^1^Department of Power Mechanical Engineering, National Tsing Hua University, Hsinchu, Taiwan

^2^National Institute of Cancer Research, National Health Research Institutes, Tainan, Taiwan

^3^Division of Hematology and Oncology, Department of Internal Medicine, National Cheng Kung University Hospital, Tainan, Taiwan

^4^Institute of Clinical Medicine, National Cheng Kung University Hospital, Tainan, Taiwan

^5^Institute of Biomedical Engineering, National Tsing Hua University, Hsinchu, Taiwan

^6^Institute of NanoEngineering and Microsystems, National Tsing Hua University, Hsinchu, Taiwan

†First author: Dr. Lien-Yu Hung; email: weslyehung@gmail.com.

‡Co-first author: Dr. Nai-Jung Chiang; email: njchiang@nhri.org.tw.

*Corresponding author: Dr. Gwo-Bin Lee; email: [gwobin@pme.nthu.edu.tw](mailto:gwobin@pme.nthu.edu.tw); Tel: +886-3-5711531 ext. 33765; Fax: +886-3-5742495

^+^Co-corresponding author: Dr. Yan-Shen Shan; email: [ysshan@mail.ncku.edu.tw](mailto:ysshan@mail.ncku.edu.tw); Tel: +886-6-2353535 ext. 3105; Fax: +886-6-2758781

1. The experimental protocol of the on-chip Cell-SELEX

Supplemental Table 1: detailed information about the experimental protocol on the Cell-SELEX microfluidic platform

| Step | Procedure | Sample volume (μL) | On-chip operation condition |
| --- | --- | --- | --- |
| 1. | Load anti-EpCAM beads and pre-treated bile into the chamber A | 10/600 |  |
|  | Load 1×PBS into the chambers P | 600 |  |
|  | Load 16% paraformaldehyde into the chamber D | 200 |  |
|  | Load 0.1% Triton X 100 into the chamber E | 200 |  |
|  | Load diluted first antibody chamber into the chamber F | 200 |  |
|  | Load diluted secondary antibody into the chamber G | 200 |  |
|  | Load diluted DAPI/Hoechst into the chamber H | 200 |  |
| 2. | Incubate anti-EpCAM beads and pre-treated bile for 15 min |  | -100 mmHg and 0.5 Hz for the micromixer |
| 3. | Collected bead-cancer cell complexes by a magnet and remove the incubation supernatant through the waste chamber |  | -500 mmHg for the suction pressure |
| 4. | Transport 1×PBS into the micromixer | 600 | -100 mmHg and 0.5 Hz for the micromixer |
| 5. | Wash the collected bead-cancer cell complexes by the micromixer for 1 min |  | -100 mmHg and 0.5 Hz for the micromixer |
| 6. | Remove the incubation supernatant through the waste chamber |  | -500 mmHg for the suction pressure |
| 7. | Repeat the steps 4-6 for another two times |  |  |
| 8. | Transport 1×PBS into the micromixer | 600 | -100 mmHg and 0.5 Hz for the micromixer |
| 9. | Incubate bead-cancer cell complexes with 4% paraformaldehyde for 3 min |  | -100 mmHg and 0.5 Hz for the micromixer |
| 10. | Collected bead-cancer cell complexes by a magnet and remove the incubation supernatant through the waste chamber |  | -500 mmHg for the suction pressure |
| 11. | Transport 1×PBS into the micromixer and wash the collected bead-cancer cell complexes by the micromixer for 1 min | 600 | -100 mmHg and 0.5 Hz for the micromixer |
| 12. | Repeat the steps 10-11 for another two times |  |  |
| 13. | Collected bead-cancer cell complexes by a magnet and remove the incubation supernatant through the waste chamber |  | -500 mmHg for the suction pressure |
| 14. | Transport 1×PBS into the micromixer | 600 | -100 mmHg and 0.5 Hz for the micromixer |
| 15. | Incubate bead-cancer cell complexes with 0.1% Triton X 100 for 3 min |  | -100 mmHg and 0.5 Hz for the micromixer |
| 16. | Collected bead-cancer cell complexes by a magnet and remove the incubation supernatant through the waste chamber |  | -500 mmHg for the suction pressure |
| 17. | Transport 1×PBS into the micromixer and wash the collected bead-cancer cell complexes by the micromixer for 1 min | 600 | -100 mmHg and 0.5 Hz for the micromixer |
| 18. | Repeat the steps 16-17 for another two times |  |  |
| 19. | Collected bead-cancer cell complexes by a magnet and remove the incubation supernatant through the waste chamber |  | -500 mmHg for the suction pressure |
| 20. | Transport 1×PBS into the micromixer | 600 | -100 mmHg and 0.5 Hz for the micromixer |
| 21. | Incubate diluted first antibody and pre-treated bile for 15 min |  | -100 mmHg and 0.5 Hz for the micromixer |
| 22. | Collected bead-cancer cell complexes by a magnet and remove the incubation supernatant through the waste chamber |  | -500 mmHg for the suction pressure |
| 23. | Transport 1×PBS into the micromixer and wash the collected bead-cancer cell complexes by the micromixer for 1 min | 600 | -100 mmHg and 0.5 Hz for the micromixer |
| 24. | Repeat the steps 22-23 for another two times |  |  |
| 25. | Collected bead-cancer cell complexes by a magnet and remove the incubation supernatant through the waste chamber |  | -500 mmHg for the suction pressure |
| 26. | Transport 1×PBS into the micromixer | 600 | -100 mmHg and 0.5 Hz for the micromixer |
| 27. | Incubate diluted secondary antibody and diluted DAPI/Hoechst pre-treated bile for 3 min |  | -100 mmHg and 0.5 Hz for the micromixer |
| 28. | Collected bead-cancer cell complexes by a magnet and remove the incubation supernatant through the waste chamber |  | -500 mmHg for the suction pressure |
| 29. | Transport 1×PBS into the micromixer and wash the collected bead-cancer cell complexes by the micromixer for 1 min | 600 | -100 mmHg and 0.5 Hz for the micromixer |
| 30. | Repeat the steps 28-29 for another two times |  |  |
| 31. | Collected bead-cancer cell complexes by a magnet and remove the incubation supernatant through the waste chamber |  | -500 mmHg for the suction pressure |
| 32. | Transport 1×PBS into the micromixer | 600 | -100 mmHg and 0.5 Hz for the micromixer |
| 33 | Collected all re-suspended bead-cancer cell complexes for further fluorescence microscopy analysis. |  |  |

1. Characterization of the micromixer/micropumps, and microvalves

The integrated microfluidic chip was consisted of three layers, including two PDMS layers and one glass substrate. The first PDMS layer was a thin layer with a thickness of 150 μm, which was a liquid channel layer. Another thick PDMS layer (around 1500 μm), which contained air channels, was used to control the micropumps.

The main function of the transportation unit of the microfluidic chip was to transport the binding buffer and the washing buffer to the target cell region. Because the transport process requires three steps, reducing the operating time for each fluid transport step could be effectively achieved by increasing the overall flow rate. The fluidic pumping rate was also found to increase with an increase in the applied gauge pressure (i.e. higher vacuum). The maximum pumping rate of the transportation unit was found to be 233.5 μL/sec when operated at a frequency of 0.5 Hz at a gauge pressure of -100 mmHg.

1. The on-chip CCA cancer cell capture protocol which required pre-processing of bile samples with spiked-in cells

The on-chip CCA cancer cell capture protocol which required pre-processing of bile samples were performed with large (2×10^5^) and small amounts (10 cells and one cell) of spiked-in cells, which presented 82.5±4.3, 70.0±8.1, and 66.6±4.7%, respectively, as shown in Supplemental Information Figure 2, Figure 3, Supplemental Information Table 2, and Table 3. It is important to note that these cell-spiked bile fluids were purified from diseased-bile samples by centrifugation since there was no non-diseased bile sample available.


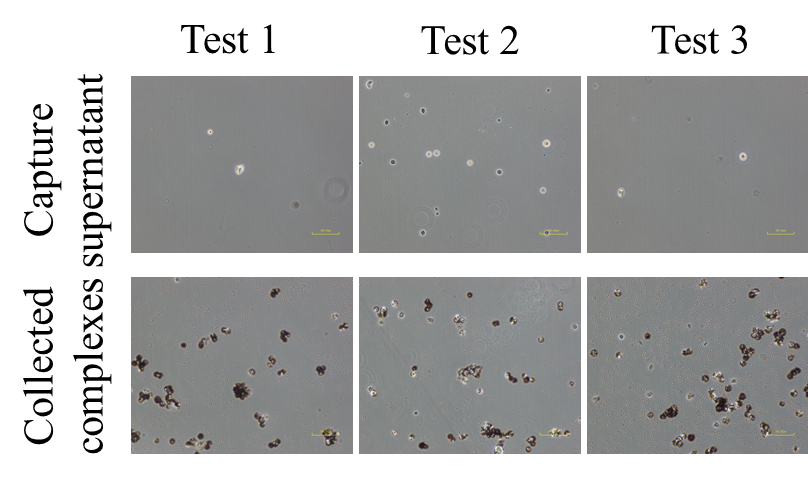


Supplemental Information Figure 1. The on-chip CCA cancer cell capture protocol which required pre-processing of bile samples was performed with a relatively large amount of spiked-in cells (2×10^5^)

Supplemental Information Table 2. Counted cells with a relatively large amount of spiked-in cells (2×10^5^)

| Spike-in cells | Test 1 | Test 2 | Test 3 | Capture rate (%) |
| --- | --- | --- | --- | --- |
| 2×10^5^ | 1.75×10^5^ | 1.70×10^5^ | 1.50×10^5^ | 82.5±4.3 |


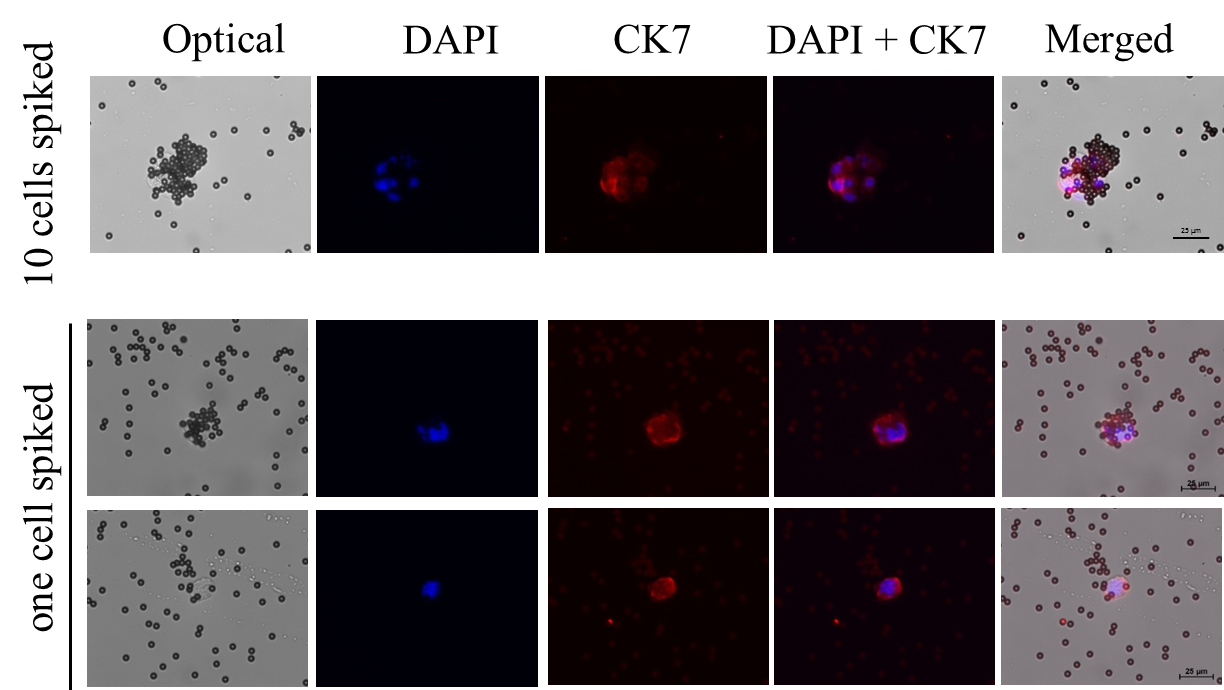


Supplemental Information Figure 2. The on-chip CCA cancer cell capture protocol which required pre-processing of bile samples was performed with a small amount of spiked-in cells (10 cells and one cell).

Supplemental Information Table 3. Counted cells with a small amount of spiked-in cells (10 cells and one cell)

| Spike-in cells | Test 1 | Test 2 | Test 3 | Capture rate (%) |
| --- | --- | --- | --- | --- |
| 10 | 6 | 8 | 7 | 70.0±8.1 |
| 1 | 1 | 1 | 0 | 66.6±4.7 |

1. The fluorescent images presented under bench-top IF staining process


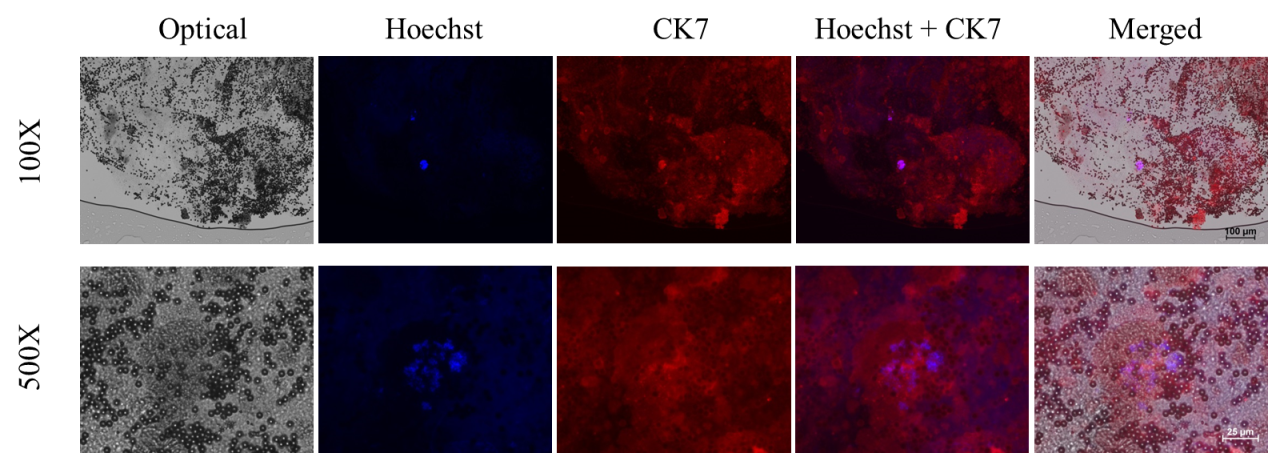


Supplemental Information Figure 3. fluorescent images presented under bench-top IF staining process.
